# Rapid diagnosis of malaria by chemometric peak-free LIBS of trace biometals in blood

**DOI:** 10.1038/s41598-022-22990-8

**Published:** 2022-11-23

**Authors:** Wayua Deborah Musyoka, Angeyo Hudson Kalambuka, Dehayem-Massop Alix, Kaduki Kenneth Amiga

**Affiliations:** Department of Physics, Faculty of Science and Technology, University of Nairobi, P. O. Box 30197-00100, Nairobi, Kenya

**Keywords:** Biomarkers, Pathogenesis, Optics and photonics, Physics

## Abstract

Laser Induced Breakdown Spectroscopy (LIBS) trace atomic species of diseased biofluids are subtle (peak-free) in complex spectra. Trace analysis requires a considerable push in analytical strategy. Enabling LIBS with chemometrics can help identify, extract, analyze and interpret the trace species’ spectral signatures to give an insight on the biophysiological status of the bodies from which the biofluids originate. We report on the trace quantitative performance of peak-free LIBS enabled by chemometrics modelling using principal components analysis (PCA) for direct artificial neural network (ANN)–based analysis of Cu, Zn, Fe and Mg in *Plasmodium falciparum*-infected blood in the context of rapid spectral diagnosis of malaria utilizing the biometals as the disease biomarkers. Only one standard is required in this method—to delineate the analyte spectral regions (feature selection) and to test for accuracy. Based on the alteration of the biometal levels and their multivariate and correlational patterns in cultured blood, peripheral finger blood drops dried directly on Nucleopore membrane filters was accurately discriminated as either malaria-infected or healthy. Further the morphological evolution of *Plasmodium* was accurately predicted using spectral features of the biometals wherein high negative correlations between Fe (− 0.775) and Zn (− 0.881) and high positive correlations between Cu (0.892) and Mg (0.805) with parasitemia were observed. During the first 96 h of malaria infection Cu increases profoundly (from 328 to 1999 ppb) while Fe, Zn and Mg decrease (from 1206 to 674 ppb), (from 1523 to 499 ppb) and (from 23,880 to 19,573 ppb) respectively. Compared with healthy, *Plasmodium falciparum*-infected blood has high Cu but low levels of Fe, Zn and Mg. Cu and Zn are highly (≥ 0.9) positively correlated while Fe and Cu as well as Zn and Cu are highly (≥ 0.9) negatively correlated. Chemometric peak-free LIBS showed the potential for direct rapid malaria diagnostics in blood based on the levels, alterations and multivariate associations of the trace biometals which are used as biomarkers of the disease.

## Introduction

Body fluids have characteristic biochemical compositions but which change in response to pathological stimuli^1^. This leads to impairment of biological functions resulting to abnormalities in body metabolism^2–4^ as well as structural changes and functionality of essential body components^5–7^. Accurate analysis, therefore of trace biometals in body fluids and tissue, particularly of those biometals involved in regulation of immunity is of interest since their levels, alterations and multivariate associations can be metrics for disease diagnosis utilizing the biometals as biomarkers. Metals and metalloids are crucial for a series of biopathological processes: up to a third of all proteins require bound metals to carry out their diverse functions as metallloproteins^8–10^. Roughly half of the enzymes use a metal cofactor to provide the catalytic, regulatory and structural roles of protein function^11^. Zn is for instance vital in the functioning of more than 300 enzymes and stabilization of the DNA structure^12^.

Nonetheless sequential changes in trace biometals in fluids harbouring infections are hardly studied. Blood trace biometals have a role in both the protection as well as exacerbation of malaria, which is now considered to be perhaps the most important parasitic disease of man^13^. Malaria is a major problem especially in the Tropics where it is a leading cause of mortality^14^. The disease arises from infestation of the red blood cells by *Plasmodium* parasites, the most common species in Africa^15,16^. The infestation causes major changes in the metabolism and transport of important cellular components: a host’s body normal response to infection includes increased synthesis of metal-binding proteins and a concomitant flux of trace elements in tissues.

For which reason the relationship of biometal levels in blood with cellular disorders linked to pathogenesis has been increasingly receiving attention^17–22^. Dogan et al.^23^ used total reflection X-ray fluorescence (TXRF) to study behcet disease in serum. Using Mössbauer spectroscopy Bauminger et al.^24^ determined the levels of Fe in serum and red blood cells in several blood diseases including malaria. Huszank et al*.*^25^ analyzed Na, P, S, Cl, K, Ca, Fe, Cu, Zn, and Br directly in ∼ 10 μL of blood. A dried blood spot method based on direct sampling by laser ablation-inductively coupled plasma-mass spectrometry (LA-ICP-MS) was reported to provide low limit of detection (LOD) and limit of quantification (LOQ) of 3.5 μg L^−1^ and 11.6 μg L^−1^, respectively^26^. Se, Cu, Zn, Br and Rb were determined in very small sample volume (0.75 μL) of human serum and mice whole blood by X-ray fluorescence (XRF)^27^ wherein accurate results were obtained without the need of exact volume measurement because the backscatter correction method was used. Most of the above techniques however require sophisticated sample preparations including addition of chemical precursors and acid digestions.

A technique which is rapid and could be used directly, needing only one drop of blood without special preparation, would be advantageous in practice. Because most malaria occurs in resource-constrained regions of the world, the technique should preferably be portable, easy to use and inexpensive. LIBS presents unique advantages in these respects as it requires little or no sample preparation and therefore saves on time; it is ideal for remote applications especially when analyzing biohazardous samples; its spectral range has many lines available for analysis; and lastly it is minimally invasive and can perform real-time, stand-off spectroscopy^27,28^. LIBS has also the advantages of small sample requirement (0.1 μg–1 mg) converted to plasma^29,30^, and high sensitivity for determination of low-Z metals for which only a few analytical methods are available. Further, hand-held LIBS systems now exist for field/ clinical deployment.

In LIBS a high energy, pulsed laser beam is focused onto the analysed sample to create a high temperature microplasma wherein the sample is ablated directly into atoms and ions. The subsequent emission becomes the analytical signal containing quantitative information from which the elemental composition can be determined. Since the precursor biochemicals in body fluids are in very low concentrations, their quantitative analysis requires to be made sensitive and accurate. The complexity of such samples (and subsequent data interpretation) constitutes a multivariate analysis problem; so chemometrics techniques may be used to overcome the challenge as they have ability to reduce the dimensionality of the spectral data and to extract the subtle biomarker information from interfering spectra without altering the crucial analytical information^31,32^. This work aimed at exploring chemometric peak-free LIBS for diagnostic analysis of trace biometal (Zn, Fe, Mg, Cu) concentrations and their multivariate alterations in human blood (embedded onto Nucleopore membrane filters) in relation to malaria onset and pathogenesis. The potential of the dried blood spot analysis has been recognized in diverse fields^33–44^. Its advantages over liquid blood analysis lie in the minimally invasive sampling, the sufficiency of small blood volumes as well as the simple transport and storage requirements^45–47^. The study focused on these biometals since they influence the cellular and molecular immunological functions, which are important in the host of *Plasmodium* parasite. Fe, Cu, Mg and Zn are also either antioxidants or antioxidant enzyme co-factors fighting against abnormal elevation of oxidative stress both from host and the parasite^48–51^. Most studies on *Plasmodium falciparum* infection for malaria diagnosis have focused on the morphological aspect of the parasite, the structure of the red blood cells and how it changes in optical environment, as well as on the by-product of red blood cells (hemozoin) ingestion by the malaria parasites^52–55^. Common methods of malaria diagnosis (optical microscopy (the ‘Gold Standard’), rapid diagnostic tests, molecular diagnosis, and symptoms-based (which are not specific to malaria)) seek to determine the presence of *Plasmodium* in the body^56^. These methods are labour-intensive, tedious, and require skilled personnel.

## Methods

### Preparation of parasite culture

*Plasmodium falciparum* parasites were cultured in a complete medium at 1% haematocrit at 37 °C in a 5% CO_2_/3% O_2_/balanced N_2_ gas mixture. The complete media, a mixture of healthy O + human blood and RPMI 1640 culture media, were used as the control samples. Infected human red blood cells were washed three times (to remove any undesired components such as bacteria) and stored at between 4 and 8 °C. They were then added to the complete media to initiate the culture process. Whole blood samples were taken from the consenting 20 malaria patients as well as 23 healthy volunteers at the Kenyatta National Referral Hospital, Nairobi, Kenya. To take part in the study, all of the participants were well briefed and they agreed to participate. They consequently all provided informed consent. The study was approved by the Kenyatta National Referral Hospital-University of Nairobi Ethics and Research Committee (ERC Certificate Number: P112/03/2018). All the methods that were used to handle and prepare samples in this study were performed in accordance with the relevant guidelines and regulations in the Ethics Research Committee certificate. The Haematocrit (initial parasitemia) was measured from a packed red blood cells volume that had been centrifuged in a swinging bucket rotor at 2000 × *g* for 5 min at room temperature. Schizonts were isolated on Percoll cushions. For RS parasite analysis, aliquots were layered on top of 70% Percoll in 1.6 mL tubes and centrifuged at 4000 × *g* for 5 min. Red blood cell pellets were then washed once with RPMI 1640 medium and immediately frozen at − 20 °C until they were used. The pellets were added to the complete media and kept in the CO_2_ incubator at 37 °C and thereafter harvested after every 24 h for four days^57^.

### Staining and examination of thick and thin smears

Each time the media was harvested both thick (6 μL) and thin (2 μL) blood smears were prepared on glass slides. The slides were immersed in absolute methanol for fixation, then dried. 10% (1:9 mL) for 10 min and 3% (3:97 mL) for 45–60 min fresh, working Giemsa stains were prepared. The stained blood smears were washed with water to remove excess stain, dried and observed under a microscope. The thick blood smear were used to microscopically confirm infection by *Plasmodium falciparum*; while the thin smears were used to determine the parasite morphology. The smear was first screened at a low magnification (10X × 40X objective lens) to detect suitable fields for analysis then later examined using X100 oil immersion. Parasitemia and morphology were determined at each harvested stage. The percent of infected red blood cells was determined by enumerating the number of infected in relation to the number of uninfected cells. This method required the preliminary determination of the number of erythrocytes present in the average microscopic field. Although in this work sub-culturing was not done and therefore the media and pellets were not renewed hence the parasitemia should be higher, the parasite load, expressed as a percentage parasitemia was in agreement with other studies where *Plasmodium falciparum* was cultured without change of media^58,59^.

### LIBS instrumentation and sample analysis

**Figure 1:**
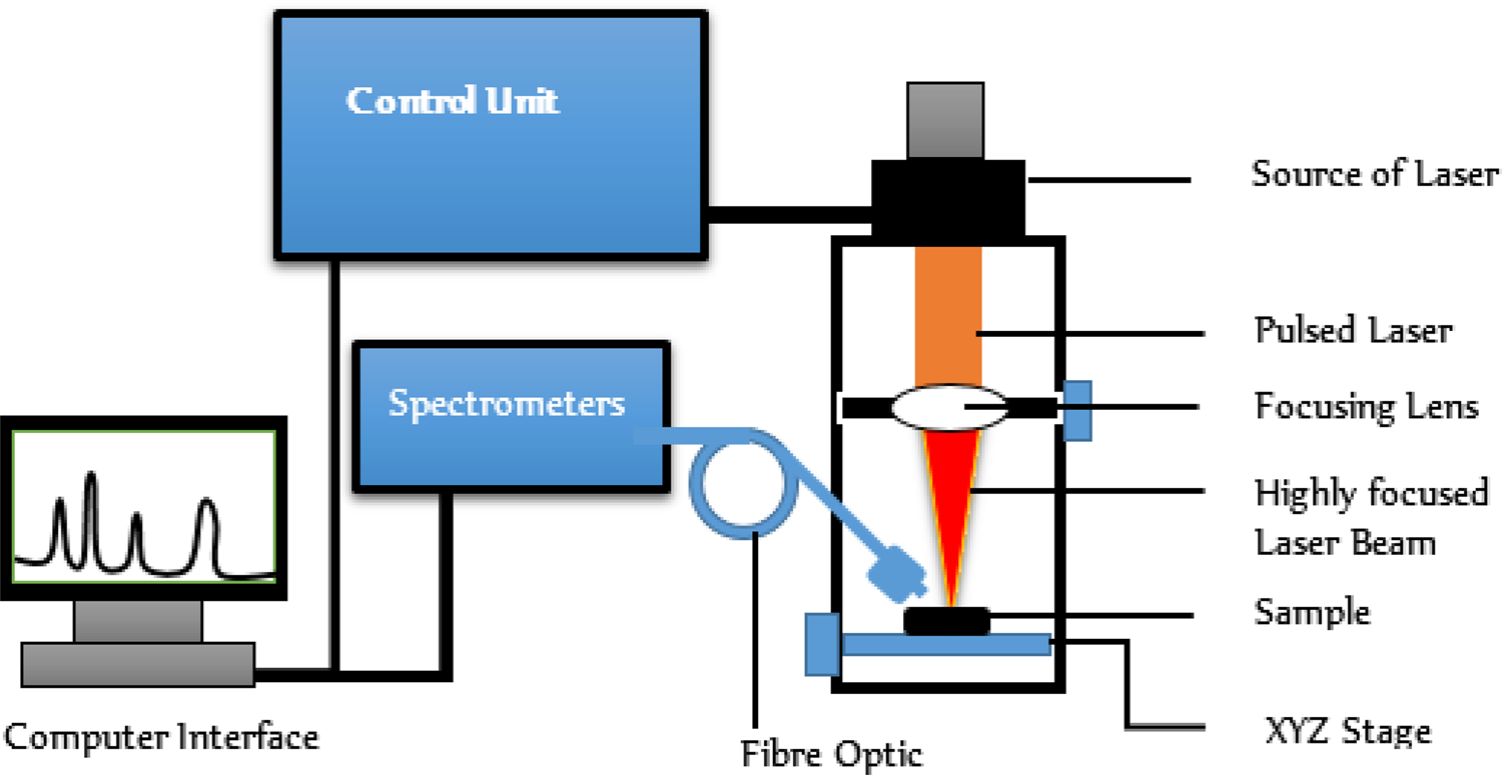
Schematic diagram of the Ocean Optics LIBS spectrometer.

The Ocean Optics LIBS2500plus spectrometer (based on CFR Nd:YAG laser, Big Sky Laser Technologies) was used in this study (Fig. 1). The laser specifications are: maximum energy per pulse = 250 mJ; operational wavelength = 1064 nm, pulse duration = 8 ns; pulse repetition frequency = 15 Hz (i.e. the period is 0.067); max. peak power = 31.25 MW). The spectrometer has a broad-band 7-CCD system spanning the wavelength range 200–980 nm at ≈ 0.065 nm resolution.

Spectrometer optimization involved systematic exploration of the signal-to-noise ratio of the analyse spectra at varying laser energies, integration time, delay time, and optical fibre-to-sample distance. The optimal values were: delay time: 0.4 μs; integration time: 150 μs. The blood samples to be analysed were placed on a manually controlled X–Y stage 8 mm away from the focal lens. The LIBS emission was transmitted to the detection system via a fused silica optical fibre (0.22 numerical aperture, focal length 101 mm)^60^. For each sample analysed, three single shot (ablation) spectra were obtained from different spots and averaged.

**Figure 2:**
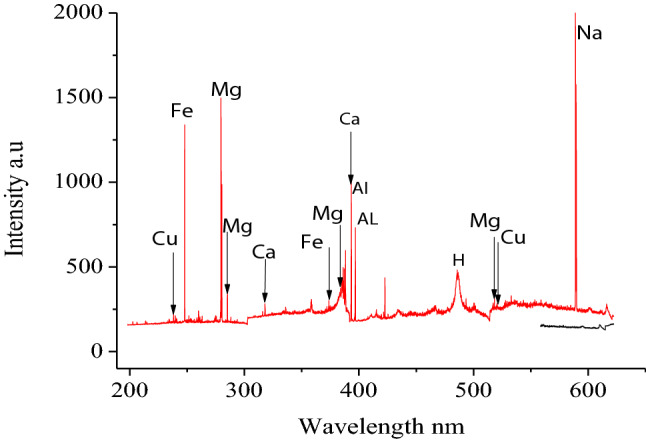
Example of LIBS spectrum of Oyster (NIST 1566B) tissue.

Figure 2 shows among others the spectral lines of Cu, Fe and Mg exited in Oyster (NIST 1566B) tissue. Spectral regions of interest (ROI) analyte signals corresponding to the lines of the trace biometals of interest were identified^61^ based on the Oyster tissue spectra.

**Figure 3:**
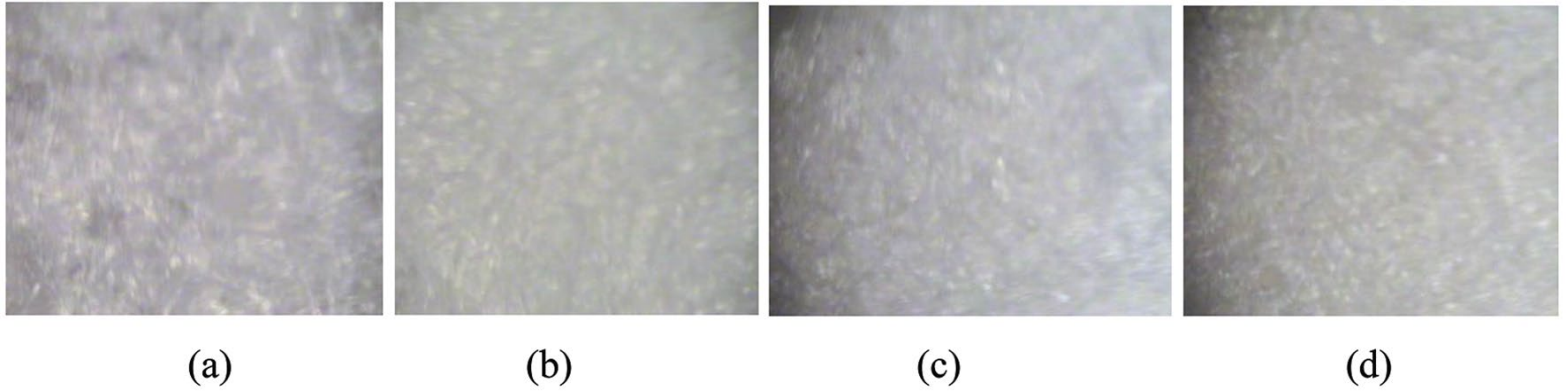
Images of (**a**) simulate blood sample (**b**) Oyster (NIST 1566B) CRM (**c**) healthy blood sample and (**d**) *Plasmodium falciparum*-infected blood sample (10X).

The images shown in Fig. 3 illustrate the surface morphology of the blood matrix samples as prepared on the Nucleopore membrane filter compared to that of an Oyster (NIST 1566B) certified reference material (CRM); the blood drops were evenly spread on the membrane filter using a micropipette and left in a sterile hood to dry to ensure the notable homogeneity (Fig. 3).

### Trace biometal analysis using artificial neural networks (ANN) calibration models

**Figure 4:**
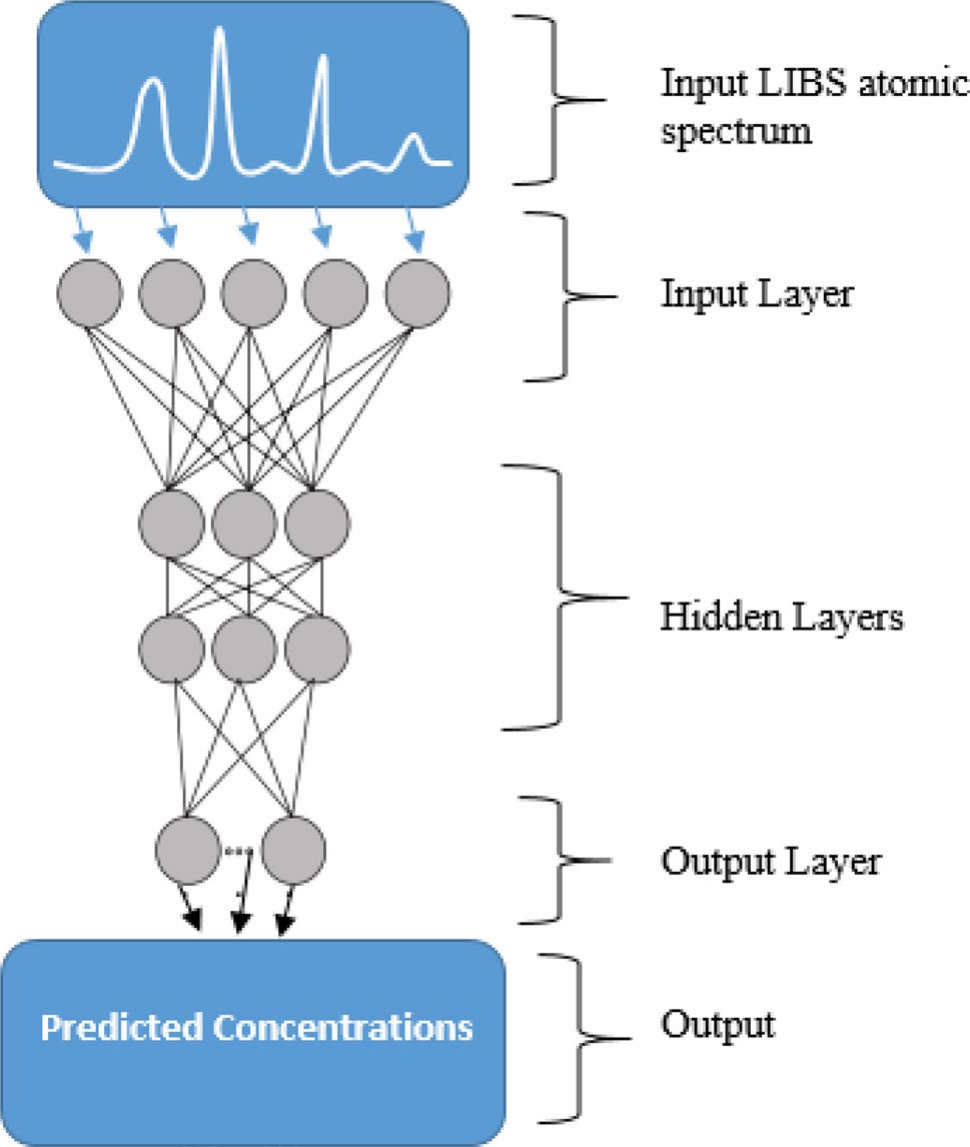
Schematic diagram of feed forward back propagation ANN^63^.

ANN is one of the computational ways of mapping non-linear input data to a target space and thus can perform functions such as pattern recognition as well as regression. The most common of the ANN network architectures is the multilayer feed-forward where the input data proceeds forward only (to the hidden layer and then to the output layer) and never makes loops as opposed to other architectures such as the recurrent system^62^. The multilayer feed-forward system consists of three layers—input, neuron transfer function, and output (Fig. 4). The power of an ANN depends on the transfer function and the learning rule^32^.

An ANN may be mathematically captured as outlined in Eq. (1):

1 $$y = f\left( {\mathop \sum \limits_{i} wix_{i} + w_{0} } \right)$$

where *y* is the output of the neuron, *x*_*i*_ is the input and *w*_*o*_ is an offset term. The value of the qth output from the network, *y*_*q*_ is given by

2 $$y_{q} = \sum \left( {x_{i} *\omega_{iq} } \right)$$

The algorithm itself can be divided into four main parts: the feed forward nature of computation, a gradual back propagation to the output layers, a continuous back-propagation to the hidden layers of the network, and a gradual update of the weights of the network.

Prior to analysis the spectra were pre-processed (via smoothing by moving average with a segment size of 3) in order to eliminate spectral data that is irrelevant to the model building. The pre-processed spectral databases were compressed into a smaller matrix representing each biometal (selected spectral features corresponding to the trace biometals of interest) and used as the model inputs. As the trace biometal spectral responses are subtle and not easily discernible with naked eye, the peak positions and respective regions of interest (ROI) for the biometals were determined based on Oyster tissue (NIST 1566B) certified reference material (CRM) in which their respective concentrations are high enough to produce discernible spectral peaks. The targets were developed with the same number of columns as the input data. The datasets were imported to the neural network platform for analysis using MATLAB version 7.8 (Mathworks).

The network randomly divides the input and output data set into three categories, that is, 60% training, 20% validation and 20% test set. The number of neurons was determined by observing the performance and regression parameters of the network for each biometal: Cu and Fe had their best performances at 3 neurons while Zn and Mg had best performances at 5 neurons.

Since a proper blood matrix-matched standard was not available in our laboratory (due to resource constraints) the developed analytical models were tested for their predictive ability using standard reference material (SRM)-1598a (inorganic constituents in animal serum). SRM-1598a is a serum sample derived from a mixture of serum from healthy bovine and porcine animals and is commonly used to evaluate the accuracy of analytical methods for selected elements in biological fluids similar to blood serum and plasma. Root mean square error of calibration (RMSEC) was used to indicate the average difference between predicted and measured values while standard error of performance (SEP) indicated the variation in the precision of predictions over several samples. The back propagation training functions with Levenberg–Marquardt algorithm was used to train the feed forward networks for non-linear regression because they lead to lower mean square errors (MSE) due to their faster rate of convergence^64^. MSE values were: Zn: 0.016; Cu: 0.026; Fe: 0.01; Mg: 0.756.

Table 1 shows the analytical accuracy of the developed ANN calibration models. The obtained results demonstrate the good accuracy of the ANN calibration strategies. The Shapiro-Wilks normality test for the ANN model-determined concentrations in the blood indicated that the spectral data used for analysis was normally distributed; that is, Fe-0.8773, Cu-0.8412, Zn-0.8571 and Mg-0.9048. Therefore the accuracy of multivariate exploratory modelling for malaria diagnostics utilizing the determined biometal levels had the potential for clinical application.

**Table 1:** Comparison of Cu, Fe, Zn, Mg by ANN and SRM-1598a concentration^57^.

	Cu	Fe	Zn	Mg
NIST/SRM-1598a [Actual value—(ppm)]	1.58 ± 0.09	1.68 ± 0.06	0.88 ± 0.02	Not given
ANN model (ppm)	1.14 ± 0.74	1.14 ± 0.32	0.88 ± 0.56	17.90 ± 3.63

### Spectral data pre-modelling by principal components analysis (PCA)

PCA is a powerful tool for overview exploratory analysis as it performs a projection of the original data which allows for the visualization of the natural clustering of the data, evaluation of class similarities as well as reasons behind the observed classes or patterns. The technique is based on the evaluation of the total variance within data such that the greatest variance by any projection of the data lies on the first coordinate, the first principal component, the second greatest variance on the second coordinate, and so on.

The original data matrix is denoted as ***X***, with *n* rows, termed ‘objects’, which correspond to the samples, and *p* columns, termed ‘variables’, which comprise the measurements made on the objects. PCA will provide an approximation of ***X*** in terms of the product of two small matrices ***T*** and ***P*** as in Eq. 3 which captures the essential data patterns of ***X*** such that

3 $${\mathbf{X}} = {\mathbf{T}}.{\mathbf{P}} + {\mathbf{E}}$$

where **T** represents the scores matrix, calculated as (*n* × ***A*)**, and **P**, the loading matrix, obtained as (*A* × *p*). ***A*** is the intrinsic dimension; that is, the number of principal components necessary to describe all the information in the data set. The scores matrix expresses the relation among the samples and shows the sample coordinates in the new system of axes. The loading matrix **P** shows the relations among the variables where in this case are the intensities at different wavelengths. **E** represents a matrix of residuals. This procedure applied to spectra helps to visualize and extract information from the data and can also be applied to show clustering of similar groups ^65^. PCA modelling used both entire spectral region and select spectral features.

## Results and discussion

### Determination of trace biometals in malarial blood

Table 2 shows the results of applying the developed ANN models to determine and compare the concentrations of the trace biometals in malaria in non-infected and malaria-infected blood. The levels of Fe and Zn decrease sharply with malaria severity in agreement with earlier observations that *falciparum* malaria decreases the serum Fe significantly at high parasitemia^65,66^. Fe is required for the formation of blood in the body as well as for the transport of oxygen from the lungs. Although the interaction between Fe and malaria is more complex and controversial^67^, as a large quantity of Fe in blood is in the form of haemoglobin, decreased Fe may be attributed to the digestion of haemoglobin by the malaria parasites. The decrease in Table 2 in the levels of Mg with malaria severity was also observed by Baloch et al.^3^ and Maitland et al.^68^.

**Table 2:** LIBS determined concentrations of Cu, Zn, Mg and Fe in malarial blood.

Hours	Fe ± 32 ppb	Cu ± 78 ppb	Zn ± 43 ppb	Mg ± 189 ppb	Cu/Zn
0	1206	328	1523	23,880	**0.215**
24 h	1206	351	1521	21,843	**0.231**
48 h	805	862	849	19,548	**1.015**
72 h	514	1846	766	21,317	**2.410**
96 h	674	1999	499	19,573	**4.006**

Significant values are in bold.

Compared to their absolute concentrations the ratio of Cu/Zn has diagnostic significance: The ratio increases progressively with parasitemia; at extreme malaria severity it is > 10 times higher than at infection. Although Ginsburg et al*.*^7^ observed that while the concentration of Fe remained constant throughout the parasite cell cycle, that of Zn increased in parallel with parasite maturation than that of uninfected red blood, this increase in the Cu/Zn ratio has also been reported earlier and may be taken to be a biomarker of malaria and perhaps other diseases as well^69^. The immunomodulatory and enzyme activity simulating properties of Zn are known^3,70^.

The absolute concentration of Cu in contrast increases profoundly with degree of *Plasmodium falciparum* infection. The evidence for this was also shown by monitoring Cu concentration changes in the parasite itself where total copper content decreased by 34% in infected erythrocytes^6^. The importance of Cu homeostasis for the developmental progression of *Plasmodium falciparum* has been confirmed by inhibition of Cu-binding proteins that regulate Cu physiology and function by associating with Cu ions^71^, providing strong evidence for a link between healthy Cu homeostasis progressions of *Plasmodium falciparum*.

### Parasite density for culture samples

From Table 3, parasitic growth rate is observed to be exponential. In *falciparum* malaria there is sequestration of erythrocytes containing mature parasites in the microcirculation. This causes microvascular obstruction and accounts for much of the pathology of severe disease^72,73^. Thus, the parasites causing pathology in severe infections are not represented directly by those counted in the peripheral blood smear; patients can have the majority of their parasites circulating, or sequestered. In the latter case, the peripheral parasitemia can be low (depending on stage of development and synchronicity).

**Table 3:** Parasite density for blood culture samples.

Sample type	Hours	Stage	Parasitemia
Whole blood	(Control)	No infection	N/A
Whole blood	24	Rings/Merozoites	15%
Whole blood	48	Merozoites/Trophozoites	18%
Whole blood	72	Trophozoites/Schizonts	26%
Whole blood	96	Trophozoites/Schizonts	40%

Pearson correlation was performed on the biometal concentrations and the results, which show the relationship between the individual biometals as well as their relationship with the level of infection (parasitemia) summarized in Table 4. It will be noted in Table 4 that Fe and Cu as well as Zn and Cu have a very high negative correlation (˃ 0.9). On the other hand, as expected, there is a very high positive correlation between Zn and Cu (˃ 0.9). With respect to parasitemia there is a high negative correlation with Fe and Zn (− 0.775, − 0.881) and high positive correlation with Cu and Mg (0.892, 0.805).

**Table 4:** Pearson correlation between Fe, Cu, Zn and Mg and parasitemia.

	Fe	Cu	Zn	Mg	Parasitemia
Fe	1				
Cu	− **0.928**	1			
Zn	**0.923**	− **0.922**	1		
Mg	0.663	− 0.611	**0.825**	1	
Parasitemia	− 0.775	**0.892**	− **0.881**	**0.805**	1

Significant values are in bold.

### Malaria diagnostics based on PCA of trace biometal spectral signatures

**Figure 5:**
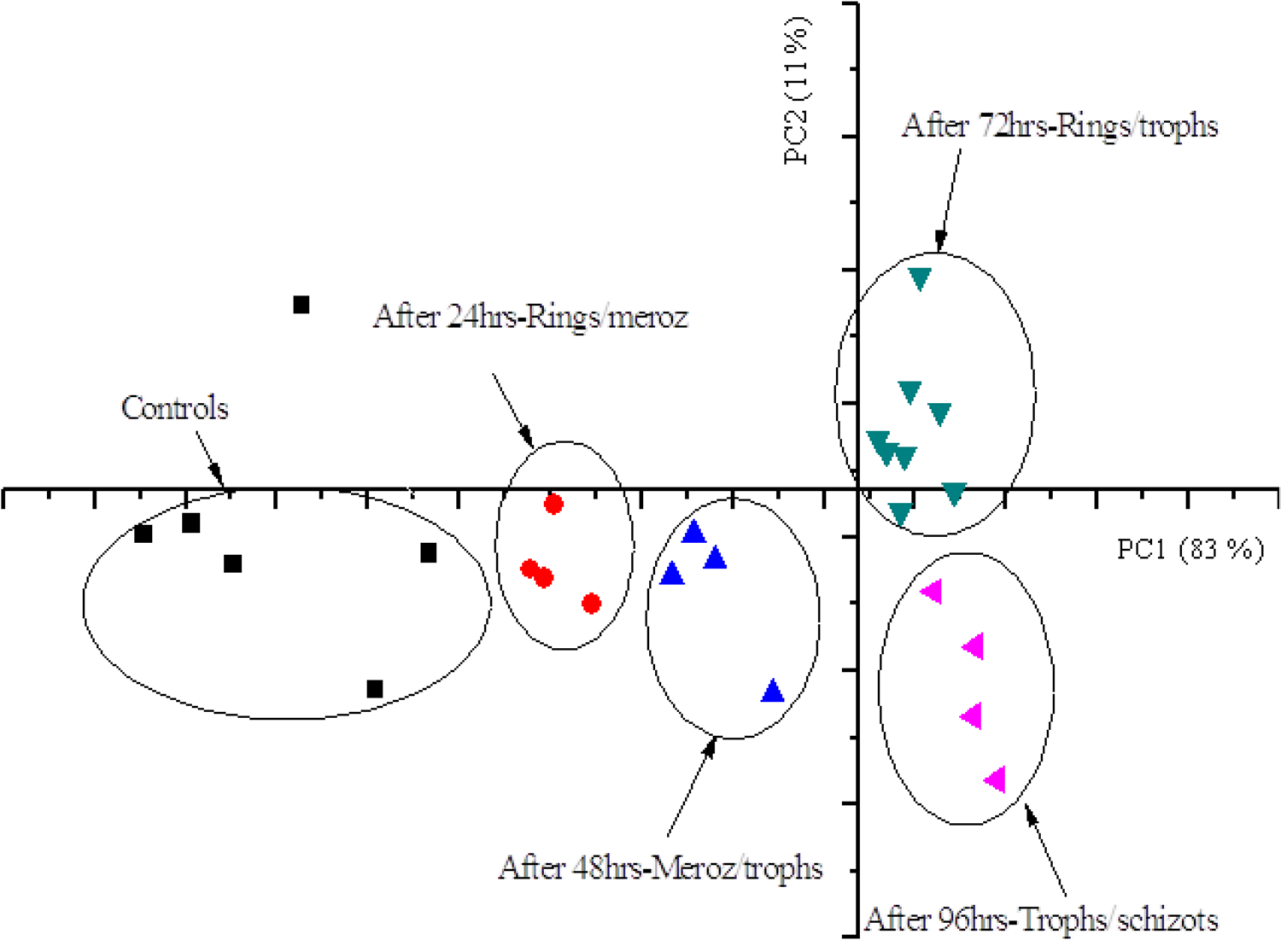
PCA score plot for cultured blood based on spectral features of the analyzed biometals.

Figure 5 shows the PCA score plot for the LIBS analysis of cultured blood infected with *Plasmodium falciparum* parasites for different degrees of infection as well as the healthy blood sample (taken here as the controls—blank). 4 PCs were observed with the first 2 PCs explaining a total variance of 94%; that is, 83% by the first PC and 11% by the second PC. It is interesting to note the observed clustering based on the degrees of severity of malaria infection. The trace biometal spectral signatures classify progressively from the initial Ring Stage of the *Plasmodium falciparum* parasite to the last stage which is characterized by Schizonts.

The samples of each group (day of harvesting) are clearly separated from each other. The early Trophozoites stage is often referred to as ‘Ring Form’ because of its morphology^74^. This enlargement is accompanied by an active metabolism that involves ingestion of the host cytoplasm and proteolysis of haemoglobin into amino acids. The end of the trophic period results to formation of Schizont, an intra-erythrocyte parasitic stage that is due to repetitive nuclear division. Merozoites bud from the mature Schizont is released following rupture of the infected erythrocyte and hence attack healthy red blood cells. Ring Stage persists for the first 18 h and thereafter the rings change to Trophozoites and Schizonts 24 h later^58^.

**Figure 6:**
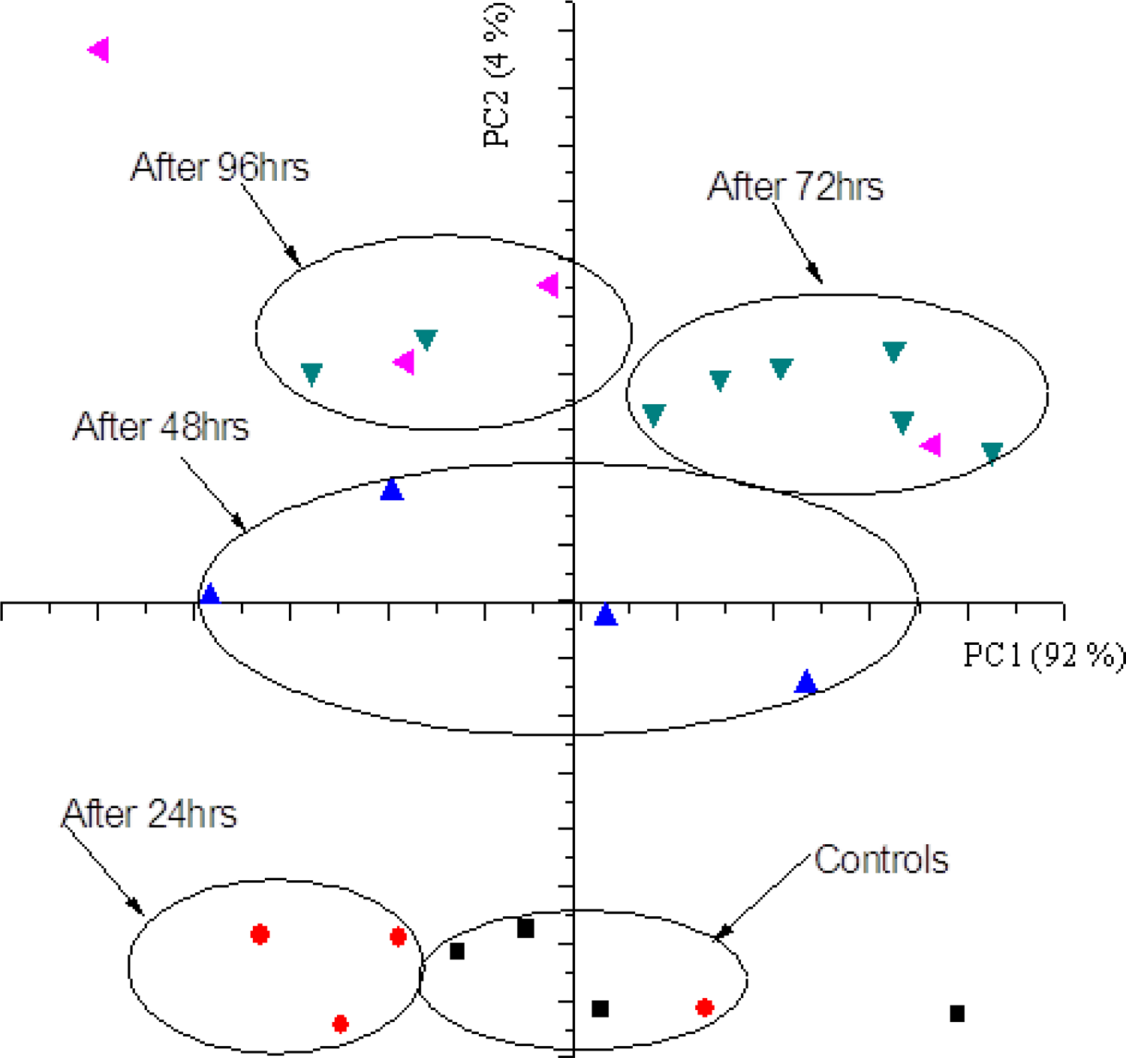
PCA score for cultured blood based on absolute concentrations of the analyzed biometals.

**Figure 7:**
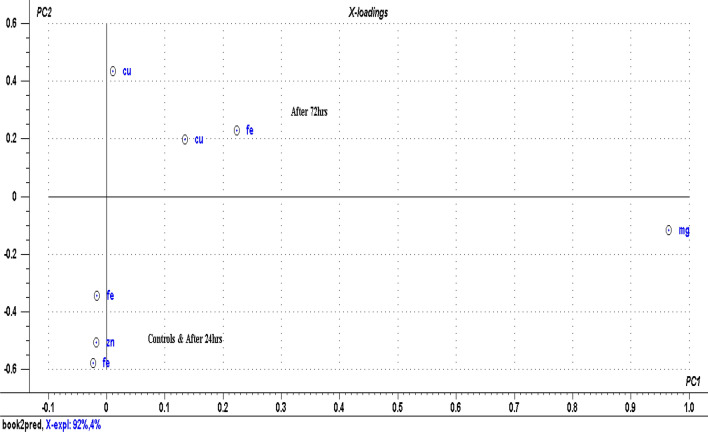
PCA loadings plot for cultured blood samples based on predicted biometal concentrations.

Figure 6 shows the score plot for cultured blood samples as well as the controls based [now] on the analyzed concentrations of the biometals as opposed to their corresponding spectral signatures. It will be noted the separation of the clusters is not as distinct as that based on biometal spectra (feature selection). Although the concentration of the biometal is the main variable in the spectral feature, LIBS spectra contain a lot of biochemical information that is also correlated to the state of disease which when appropriately extracted, analyzed and interpreted can give additional insight on the biophysiological status of the sample. We may therefore refer to these as trace biometal-mediated biochemical alterations in malaria pathogenesis. The score plot has a total explained variance of 96% with PC1 describing 92% of the total data and PC2 describing 4% of the remaining data as (also) indicated in the loadings plot Fig. 7.

The corresponding loadings plot is shown in Fig. 7 which explains the importance of each biometal in the scores groupings. For instance information after 24 h is characterized by high levels of Fe and Zn while information after 72 and 96 h is characterized by high levels of Cu. Khoshmanesh et al*.*^75^ also detected different stages of the malaria parasite, including Ring and Gametocyt based on trace element concentrations. The earlier observed high negative correlations of Cu with Fe and Zn are clearly also seen on the scores plot. It is noted that the controls and the spectral data after 24 h of infection with *Plasmodium falciparum* had predominantly high levels of Zn and Fe. The levels of Cu are also high after 72 h and 96 h with comparatively low levels of Zn.

It must be emphasized that PCA is not a classification method. PCA is an unsupervised technique, not requiring any prior knowledge of classes; the cluster formation is based only on probable pattern(s) recognition among the input dataset. Here it was utilized to have an overview of any relations in the spectral data in reduced dimension and for extraction of information (variance) from large data sets for exploratory analysis and prospective predictive modelling so as to visualize vital hidden information to inform diagnosis. To further analyze the PCA results to obtain additional information, the loadings for each PC were constructed, which, basically, represent the statistical weights of each spectral feature for the corresponding PC which represent the relation between the principal components and the LIBS spectroscopic data^76^.

**Figure 8:**
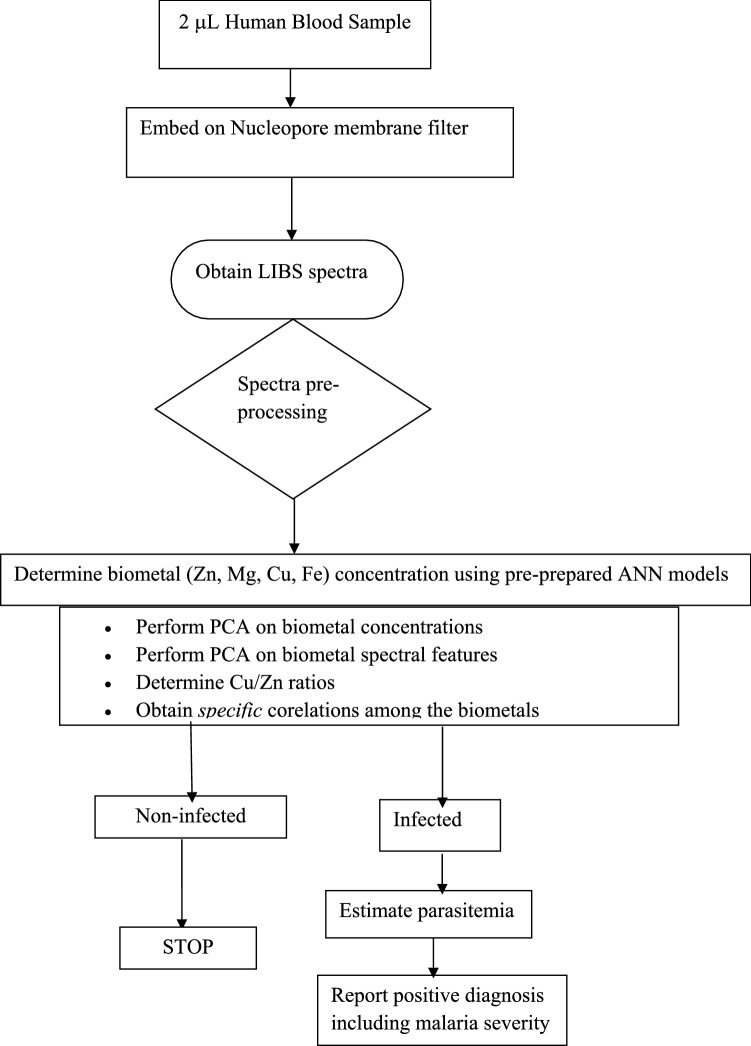
Flow chart of malaria diagnostics by chemometric peak-free LIBS.

In the flow chart (Fig. 8) it is proposed a proof-of-concept model for malaria diagnostics by chemometric peak-free LIBS of blood. Prospects are envisaged towards improvements and adaptability as well as integration of the model scheme into small, automated micro-total analysis systems. The advantage of sampling only small blood volumes could be preserved by combining the use of capillary sampling systems with dried spots of blood.

Towards this however further studies based on clinical samples are warranted to determine the malarial stage sensitivity and influence of genotype on the accuracy of diagnosis. A good diagnostic modality must have high sensitivity and be easily affordable in the tropics. The model should be applicable to all species of malaria by identifying which ones are present, provide no false positives, and it should be independent of previous malarial infections^77^.

## Conclusions

We have explored and demonstrated the utility of chemometric peak-free LIBS to perform direct trace quantitative and explorative analysis of Cu, Zn, Fe and Mg in *Plasmodium falciparum* infected blood in a manner that is applicable to rapid spectral diagnosis of malaria utilizing the biometals as disease biomarkers. In this technique there is neither cell counting nor chemical treatment; and sample preparation is minimal. Since the determined trace biometal concentrations were very low and analyte spectral peaks could not easily be observed, oyster tissue spectrum was used to identify the spectral regions of interest with which multivariate chemometrics (ANN) calibration models were developed and applied for trace biometal analysis. Using chemometrics peak-free LIBS, quantitative analysis of the biometals directly in tiny (~ μL) blood dried spots was shown to be feasible. The results indicate that the concentrations of Cu, Fe, Zn and Mg in blood alter predictably during malaria pathogenesis (onset and progression). Cu/Zn ratio was shown to be an indicator of oxidative stress during *falciparum* malaria. PCA classified the biometal profile data based on the degree of malaria infection as well as the stages of the parasites. The morphological evolution of *Plasmodium falciparum* was also accurately predicted using PCA of LIBS spectral features of the biometals. Chemometric peak–free LIBS is therefore a potentially robust technique for direct rapid malaria diagnostics in blood, a tool in which the analysis time is reduced to < 2 min/sample.

## Data Availability

The datasets used and/or analyzed during the current study are available from the corresponding author on reasonable request.

## Abbreviations

LIBS: Laser Induced Breakdown Spectroscopy
LA-ICP-MS: Laser Ablation Inductively Coupled Plasma–Mass Spectrometry
XRF: X-ray Fluorescence
CCD: Charge Coupled Detector
ANN: Artificial Neural Network
SRM: Standard Reference Material
PCA: Principal Components Analysis
TXRF: Total Reflection X-ray Fluorescence
